# Selective Non-Steroidal Glucocorticoid Receptor Agonists Attenuate Inflammation but Do Not Impair Intestinal Epithelial Cell Restitution *In Vitro*

**DOI:** 10.1371/journal.pone.0029756

**Published:** 2012-01-25

**Authors:** Kerstin C. Reuter, Stefan M. Loitsch, Axel U. Dignass, Dieter Steinhilber, Jürgen Stein

**Affiliations:** 1 Institute of Pharmaceutical Chemistry, Goethe University Frankfurt/Main, Campus Riedberg, Frankfurt/Main, Germany; 2 Department of Medicine I, Markus Hospital, Frankfurt/Main, Germany; 3 Department of Internal Medicine, Elisabethen Hospital, Frankfurt/Main, Germany; 4 Crohn Colitis Centrum Frankfurt, Frankfurt/Main, Germany; Catholic University Medical School, Italy

## Abstract

**Introduction:**

Despite the excellent anti-inflammatory and immunosuppressive action of glucocorticoids (GCs), their use for the treatment of inflammatory bowel disease (IBD) still carries significant risks in terms of frequently occurring severe side effects, such as the impairment of intestinal tissue repair. The recently-introduced selective glucocorticoid receptor (GR) agonists (SEGRAs) offer anti-inflammatory action comparable to that of common GCs, but with a reduced side effect profile.

**Methods:**

The *in vitro* effects of the non-steroidal SEGRAs Compound A (CpdA) and ZK216348, were investigated in intestinal epithelial cells and compared to those of Dexamethasone (Dex). GR translocation was shown by immunfluorescence and Western blot analysis. Trans-repressive effects were studied by means of NF-κB/p65 activity and IL-8 levels, trans-activation potency by reporter gene assay. Flow cytometry was used to assess apoptosis of cells exposed to SEGRAs. The effects on IEC-6 and HaCaT cell restitution were determined using an *in vitro* wound healing model, cell proliferation by BrdU assay. In addition, influences on the TGF-β- or EGF/ERK1/2/MAPK-pathway were evaluated by reporter gene assay, Western blot and qPCR analysis.

**Results:**

Dex, CpdA and ZK216348 were found to be functional GR agonists. In terms of trans-repression, CpdA and ZK216348 effectively inhibited NF-κB activity and IL-8 secretion, but showed less trans-activation potency. Furthermore, unlike SEGRAs, Dex caused a dose-dependent inhibition of cell restitution with no effect on cell proliferation. These differences in epithelial restitution were TGF-β-independent but Dex inhibited the EGF/ERK1/2/MAPK-pathway important for intestinal epithelial wound healing by induction of MKP-1 and Annexin-1 which was not affected by CpdA or ZK216348.

**Conclusion:**

Collectively, our results indicate that, while their anti-inflammatory activity is comparable to Dex, SEGRAs show fewer side effects with respect to wound healing. The fact that SEGRAs did not have a similar effect on cell restitution might be due to a different modulation of EGF/ERK1/2 MAPK signalling.

## Introduction

Glucocorticoids (GCs) represent one of the most powerful therapeutics available for the treatment of acute inflammation, and are a mainstay of therapy in IBD patients [1], [2]. However, the desirable anti-inflammatory and immunosuppressive properties are often accompanied by severe, and sometimes irreversible, side effects, such as fat redistribution, osteoporosis, growth suppression, diabetes, hypertension and a detrimental effect on tissue repair [3], [4]. The effects of GCs are mediated by the glucocorticoid receptor (GR), which rests inactive in the cytoplasm as a multiprotein complex containing several heat-shock proteins (Hsp), such as Hsp90 and Hsp56, (co-)chaperones and immunophilins [5], [6]. In response to ligand binding, the GR adopts an altered conformation and translocates into the nucleus, where it regulates gene expression via several mechanisms [6], [7]. Directly by binding of a ligand-GR dimer to specific DNA sequences within genes, termed glucocorticoid response element (GRE), or indirectly by interaction of a ligand-GR monomer with transcription factors such as nuclear factor κB (NF-κB), cAMP-responsive-element binding protein (CREB), activator protein (AP)-1 or signal transducers and activators of transcription (STATs) [8]. It has been hypothesised that negative gene-regulation, referred to as trans-repression, accounts for the anti-inflammatory action of GCs, whereas positive regulation, or trans-activation, contributes to some adverse effects [9], [10]. Thus, a promising new therapeutic approach based on the selective modulation of GR action and a new class of synthetic agents, the selective GR agonists (SEGRAs), aims to combine anti-inflammatory action with simultaneous reduction of adverse effects [9] [11], [12]. Along with several others, Compound A (CpdA) a plant-derived phenyl aziridine precursor isolated from a Namibian shrub [13] and ZK216348 [14], both non-steroidal in structure but exhibiting a strong preference for GR-binding, have been classified as SEGRAs and found to dissociate between trans-activation and trans-repression, both *in vitro* and *in vivo* [14], [15], [16], [17].

In the context of IBD, one of the major consequences of GC use, is the inhibition of intestinal wound healing [18], [19], [20], which limits their therapeutic application considerably, despite their excellent anti-inflammatory action. The mucosal lining of the intestine consists of fast-renewing epithelial cells which function as a barrier between the luminal environment and the mucosal immune system. In the course of IBD, damage and impairment of the intestinal epithelial surface are frequently observed, and dysfunction of the epithelial barrier results in systemic penetration of detrimental substances, leading to a generalised immune response and chronic intestinal inflammation [21], [22]. Normally, after injury, tissue integrity is restored by a rapid, organised series of cellular events in which where inflammation, cell migration and proliferation, the production of connective tissue ground substances, angiogenesis and wound contraction are orchestrated by biochemical substances [20], [23]. Furthermore, over the past decade, a complex network of regulatory peptides (chemokines, cytokines, growth factors, enzymes and extra-cellular matrix molecules) have been found to be expressed by, and to produce functional effects among, different cell populations in the mucosa [22], [24], [25], [26] contributing to the preservation of the intestinal barrier following injury. Several of these regulatory peptides, such as transforming growth factor (TGF)-β, epidermal growth factor (EGF), tumor necrosis factor (TNF)-α, hepatocyte growth factor (HGF) and insuline-like growth factors (IGF) I and II have been identified as strong healing factors, and thus play an important role in intestinal healing [22], [27], [28]. GCs' inhibition of tissue repair is currently attributed to their modulation of the wound repair processes, i.e. migration, proliferation and differentiation of epithelial cells, but in addition, to their ability to influence the expression and respective signalling pathways of a broad number of these regulatory peptides [3], [19], .

Prerequisites of disease remission in IBD are repair of the damaged epithelium and the absence of inflammation, so that normal homeostasis of the host is restored. Therefore, in the present study, the anti-inflammatory actions of the novel SEGRAs CpdA and ZK216348 in comparison to the common GC Dex were investigated in intestinal epithelial cell lines. Furthermore, with the aid of an *in vitro* wound healing model, their influences on intestinal epithelial wound repair, a key process impaired under GC treatment in IBD, and on the TGF-β and EGF/ERK1/2/MAPK signalling pathway, were studied to reveal potential causes for differences in intestinal wound healing under GC versus SEGRA deployment.

## Results

### Effects of CpdA and ZK216348 on nuclear translocation of GR

**Figure 1:**
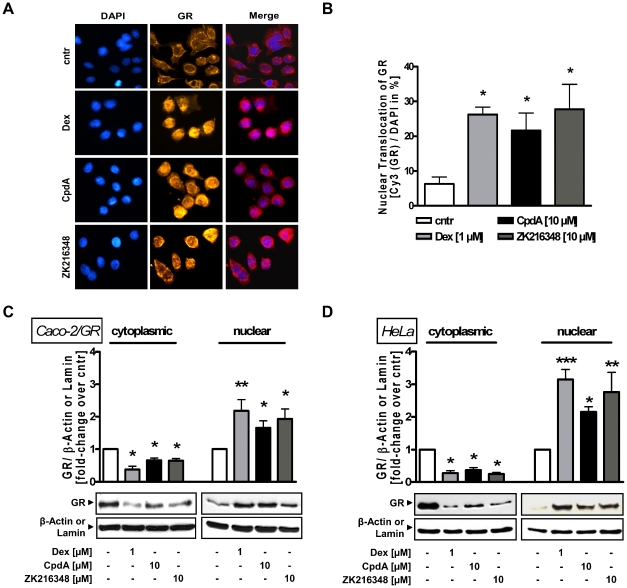
Effect of SEGRAs on GR binding and nuclear translocation. (**A**) Immunofluorescence analysis for GR location in Caco-2 cells. Cells were treated for 3 h with Dex [1 µM], CpdA or ZK216348 [10 µM]. DAPI staining was used for visualisation of the cell nuclei. (**B**) 100 cells were randomly chosen, analysed and the percentage of nuclear GR calculated. Western blot analysis for translocation of GR in (**C**) Caco-2/GR and (**D**) HeLa cell lysates after 3 h cultivation with Dex or SEGRAs. One representative blot of three is shown. Densitometric analysis of GR is normalised to β-actin (cytoplasmic extract) or lamin (nuclear extract), respectively. Bars represent mean ± S.E.M., n = 3, *P<0.05; **P<0.01 relative to vehicle.

GCs easily diffuse through the cell membrane to interact with the GR, thereby inducing its activation and subsequent nuclear translocation. To study the impact of SEGRAs on nuclear translocation of GR in colon cells, the localisation of the GR was investigated using immunofluorescence analysis. Immunostaining of non-GR-transfected Caco-2 cells revealed that both CpdA and ZK216348 induced GR nuclear translocation similar to that induced by Dex (Figure 1A and B). This was further confirmed by Western blot analysis, where in the presence of Dex and SEGRAs, the GR was predominantly localised in the nucleus of Caco-2/GR cells, indicating Dex- and SEGRA-binding to, and activation of, the GR. As described earlier for LNCaP-GR cells [17], nuclear import in the presence of CpdA was found to be reduced compared to Dex - an effect related rather to the substance CpdA than to the class of SEGRAs in general, as ZK216348 induced GR shuttling comparable to Dex. Furthermore, the reduced translocation in the presence of CpdA is not cell line specific, as the same observation was made in HeLa cell lysates (Figure 1C and D). Thus, CpdA and ZK216348 can be confirmed to be functional GR agonists in Caco-2 cells.

### Effects of CpdA and ZK216348 on GR-mediated transcriptional suppression (trans-repression)

**Figure 2:**
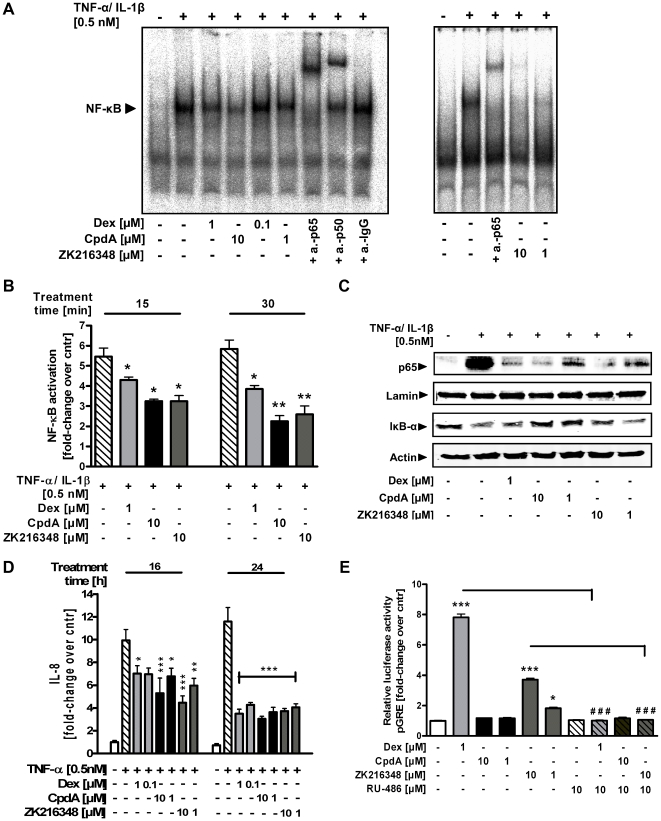
Effect of SEGRAs on transcriptional activity of GR. Cells were pre-treated with Dex [0.1–1 µM], CpdA or ZK216348 [1–10 µM] for 1 h before cultivation in the co-presence or absence of TNF-α/IL-1β [0.5 nM] and harvested for cytoplasmic and nuclear protein extract preparations. (**A**) The activity of NF-κB in nuclear extracts of IEC-6 cells was analysed by EMSA using an end-labelled NF-κB consensus oligonucleotide. The EMSA is representative for two experiments giving similar results. (**B**) NF-κB activity in nuclear extracts of Caco-2 cells was measured by transcription factor assay for p65. (**C**) Western blot analysis for nuclear p65, or cytoplasmic Iκ-Bα expression in TNF-α-/IL-1β-stimulated Caco-2 cell extracts. One representative blot of three is shown. (**D**) IL-8 content of cell culture supernatants was quantified by ELISA. Caco-2/GR cells were pre-treated with Dex [0.1–1 µM], CpdA or ZK216348 [1–10 µM] for 1 h before cultivation for 16–24 h in the co-presence or absence of TNF-α/IL-1β [0.5 nM]. Bars indicate mean ± S.E.M., n = 4, *P≤0.05; **P≤0.01; ***P≤0.001 relative to TNF-α/IL-1β treatment. (**E**) Relative Luciferase Activity of Caco-2/GR cells transfected with the glucocorticoid response element (GRE)-driven luciferase construct (pGRE-luc) and pSV-40 Renilla after 24 h of treatment with or without Dex [1 µM], CpdA or ZK216348 [1–10 µM] or co-presence of the GR agonist RU-486 [10 µM] given as 1 h pre-treatment. Bars indicate mean ± S.E.M., n = 3, ***P≤0.001 relative to vehicle, ^###^ *P*≤0.001 relative to Dex- or ZK216348-treatment, respectively.

After activation and translocation of the GR to the nucleus, the GR-mediated mechanism of trans-repression is constituted by inhibition of activation of various transcription factors and accounts for the beneficial anti-inflammatory effects of GCs. Therefore the effect of Dex, CpdA and ZK216348 was evaluated on the activity and expression of NF-κB, a key regulator of inflammation, in intestinal epithelial cells. EMSA analysis with a NF-κB consensus oligonucleotide revealed the binding of a TNF-α/IL-1β-inducible complex in nuclear extracts of IEC-6 cells after 30 min of cytokine treatment. This complex contained mainly the p65 subunit and less p50 subunit, as it was strongly supershifted by anti-p65-, and less strongly by anti-p50-specific antibody. Treatment with TNF-α/IL-1β plus Dex, CpdA or ZK216348 repressed the DNA-binding activity of NF-κB concentration-dependently, indicating an inhibitory effect of Dex and SEGRAs on transcription factor activity (Figure 2A). To exclude the possibility of cell line-specific effects, the activation of NF-κB in nuclear extracts of TNF-α-/IL-1β-stimulated Caco-2 cells was determined using the TransAM NF-κB p65 kit. Upon cytokine stimulation, a strong induction of p65 activation was observed, which was significantly suppressed by Dex and both SEGRAs (Figure 2B).

Next, it was investigated whether the Dex- and SEGRA-induced changes in NF-κB-DNA binding were associated with a reduced nuclear translocation of NF-κB. Cytokine treatment of Caco-2 cells was followed by the appearance of p65 protein within the nuclear extracts, whereas Dex and SEGRA treatment concentration-dependently decreased nuclear p65 expression. Furthermore, in contrast to results seen with TNF-α/IL-1β treatment alone, the presence of cytosolic IκB-α (inhibitor protein of NF-κB) was preserved in Dex, CpdA and ZK216348 treatment, suggesting that the decrease in nuclear p65 protein apparent with Dex and SEGRA is paralleled by an attenuated degradation of IκB-α (Figure 2C). The expression of cytokines involved in the inflammatory process has been shown to be inhibited by GCs via suppression of the activity of various transcription factors. Interleukin-8 (IL-8) represents a classically NF-κB-regulated cytokine, and it was thus evaluated whether SEGRAs were able to suppress TNF-α-/IL-1β-stimulated IL-8 secretion. Indeed, similar to those treated with Dex, Caco-2/GR cells stimulated with TNF-α/IL-1β showed a concentration-dependent decrease of cytokine-induced IL-8 secretion when treated with CpdA or ZK216348 (Figure 2D). These data indicate that, following inhibition of NF-κB activation and nuclear translocation, the tested SEGRAs exert anti-inflammatory action comparable to that of Dex.

### Effect of CpdA and ZK216348 on GR-mediated transcriptional activation (trans-activation)

In contrast to trans-repression, the mechanism of trans-activation of target gene expression by the GR-ligand complex is achieved by binding to DNA consensus sequences (GC response elements (GREs)). This is thought to be a contributing factor in the numerous side effects of GCs, as the expression of proteins involved in metabolic processes is increased by this mechanism. Therefore, with the aid of a reporter gene assay, the ability of SEGRAs to induce luciferase activity of a GRE-driven promoter construct (pGRE-Luc) transiently transfected to Caco-2/GR cells was studied. Treatment with Dex resulted in an eight-fold increase in luciferase activity, which was completely abolished by pre-treatment with RU-486, a non-selective GR and PR antagonist. This strong induction was not observed either of the SEGRAs. Although, interestingly, CpdA revealed no effect in tested concentrations, ZK216348 treatment led to the concentration-dependent acceleration of luciferase expression. This could be explained by the binding affinity of ZK216348 to other nuclear receptors, such as progesterone (PR) or mineralcorticoid receptor (MR) [14], capable of inducing luciferase response by binding to the GRE motif. This assumption is underscored by the fact that the ZK216348-effect is absent in the presence of RU-486 (Figure 2E). Moreover, in Caco-2/GR cells treated with CpdA or ZK216348, the expression of MAPK phosphatase (MKP-1) and Annexin-1, which expression is known to be initiated by the GR-mediated trans-activation mechanism, was found to be significantly reduced compared to Dex (Figure 6D and E). These results suggest that in the presence of the GR ligands, CpdA and ZK216348, the induction of gene transcription via the trans-activation mechanism is less pronounced than with Dex.

### Apoptotic and cytotoxic effects of CpdA and ZK216348 in Caco-2 and IEC-6 cells

**Figure 3:**
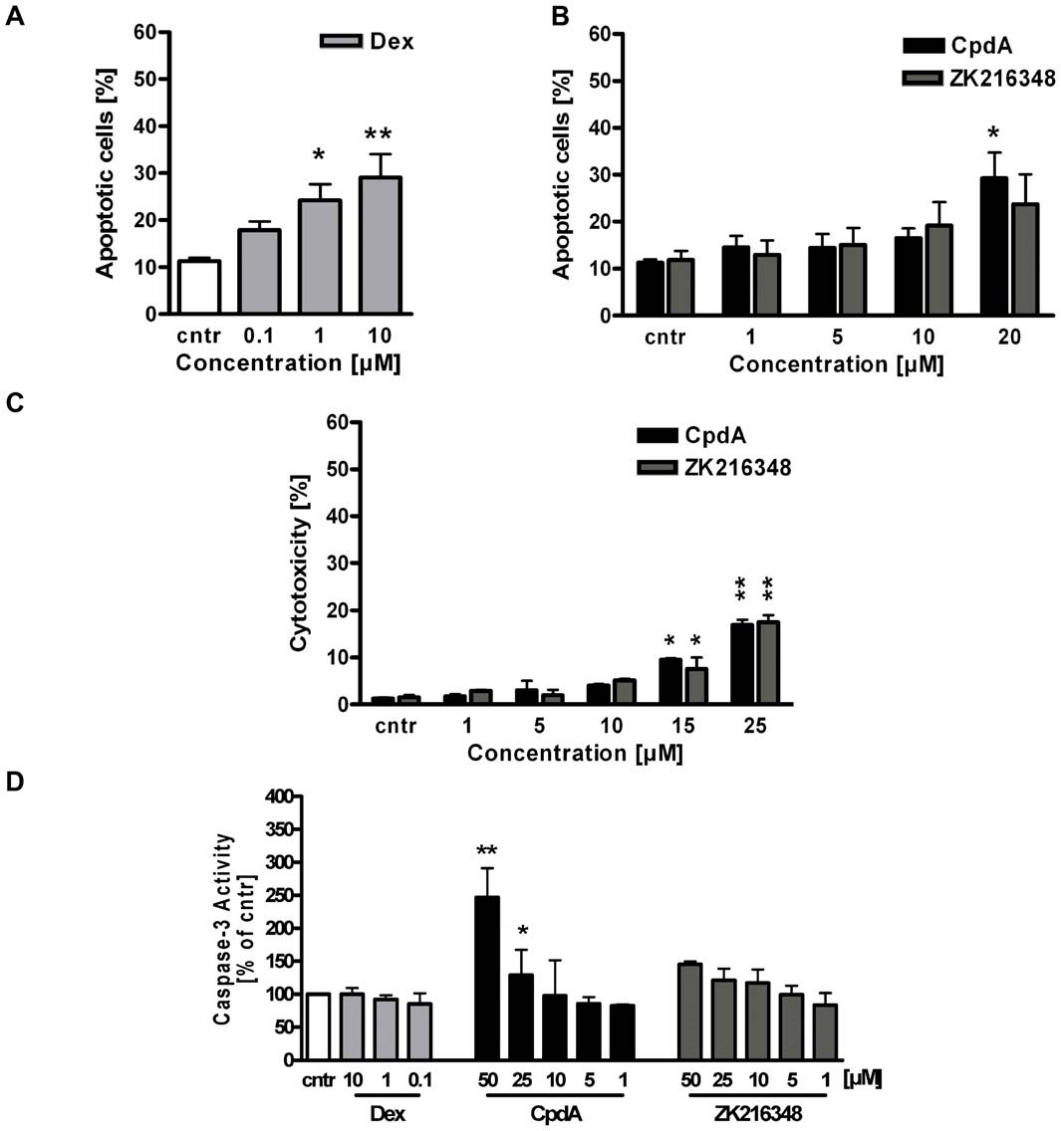
Effect of SEGRAs on cell apoptosis. Apoptotic effects of (**A**) Dex- [0.1–10 µM] or (**B**) CpdA- and ZK216348- [1–20 µM] treatment on IEC-6 cells was evaluated after 24 h by Annexin-/7-AAD staining and flow cytometric analysis. (**C**) Cytotoxicity of CpdA or ZK216348 [1–25 µM] treatment on Caco-2 cells was tested by Cytotoxicity Detection (LDH release) kit. Bars indicate mean ± S.E.M., n = 3, *P≤0.05, **P≤0.01, ***P≤0.001 relative to vehicle. (**D**) Activation of Caspase-3 in Caco-2 cells after 24 h of incubation with Dex, CpdA and ZK216348 [1–25 µM]. Results are expressed as a percentage of control. Bars indicate mean ± S.E.M., n = 3, *P≤0.05, **P≤0.01, ***P≤0.001 relative to vehicle.

Apoptosis is a central mechanism for the maintenance of homeostasis and it has already been shown that GCs have a regulative function in the apoptotic mechanisms of certain cell types [30]. Furthermore, the findings of Wuest et al. [31] and our *in vivo* experiments in the mouse model of acute TNBS-mediated colitis (unpublished data) have shown CpdA to have a narrow therapeutic window. The proven profound toxicity in higher doses was attributed to the formation of highly reactive breakdown products (aziridines) of CpdA and from these following subsequent apoptotic and cytotoxic potential. For this reason, the apoptotic effects of CpdA and ZK216348 were assessed *in vitro* in the intestinal epithelial cell lines used for our experimental setup. After 24 h treatment of IEC-6 cells with Dex, a concentration-dependent increase of apoptosis could be confirmed by FACS analysis (Figure 3A). The treatment of cells treated with ZK216348 at concentrations of 1–20 µM showed no effect on cell apoptosis; while CpdA concentrations ≥15 µM induced cell death (Figure 3B). The results of cytotoxicity testing in Caco-2 cells were in line with the FACS analysis for IEC-6 cells, where LDH activity in presence of increasing CpdA or ZK216348 concentrations was not elevated until SEGRA concentrations of ≥15 µM (Figure 3C). Similarly, the effector Caspase-3 was activated 24 h after stimulation with CpdA concentrations higher than 15 µM, while neither CpdA <15 µM nor increasing Dex or ZK216348 concentrations showed significant changes in Caspase-3 activation (Figure 3D). Taken together, evidence points to a narrow therapeutic window of CpdA and its pro-apoptotic and cytotoxic effects in higher concentrations complicates the evaluation and comparison of GR-mediated trans-activation and trans-repression effects in a wider range of concentrations (see Figure S1). Nevertheless, in Caco-2/GR cells after exposure to 10 µM CpdA or ZK216348, the concentration in which anti-inflammatory effects comparable to the common GC Dex are observed, no significant apoptosis induction, cytotoxicity or increased Caspase-3 activity compared to non-treated cells occur, indicating that the relevant effective SEGRA concentrations utilized in our experiments were not affecting cell viability of intestinal epithelial cells.

### Effects of CpdA and ZK216348 on intestinal epithelial cell restitution

**Figure 4:**
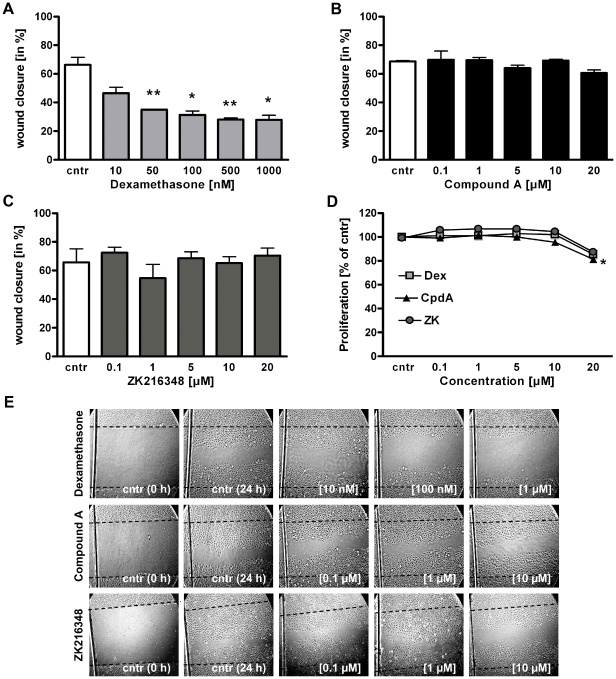
Effect of SEGRAs on intestinal epithelial cell restitution and proliferation. IEC-6 cells were wounded and cultured in the presence of (**A**) Dex [0.1–1 µM] or (**B**) CpdA or (**C**) ZK216348 [1–20 µM] for 24 h. Cell migration was assessed using an *in vitro* migration assay. Bars indicate mean values of remaining wounded area ± S.E.M., n = 3, *P≤0.05, **P≤0.01 relative to control. (**D**) BrdU incorporation assay was used for determination of IEC-6 cell proliferation after 24 h incubation with Dex or SEGRAs [1–20 µM]. Results indicate mean ± S.E.M., n = 3, ***P≤0.001 relative to vehicle. (**E**) Representative experiments illustrating the effects of Dex and SEGRAs on intestinal epithelial cell restitution. Cntr (0 h) represents cells immediately after wounding, picture cntr (24 h) and others show wounds 24 h after cultivation with Dex, CpdA or ZK216348. Dotted line indicates original margin of wound (Magnification ×100).

GCs have been shown to impair intestinal wound healing, thus preventing the full restoration of normal host homeostasis [18], [19], [20]. Usually, after superficial injury, the first step in mucosal healing involves the migration of epithelial cells across the mucosal defect to the wound area, a process termed restitution. Migration is followed by proliferation, to increase the cell pool available for wound resurfacing. To determine the effects of Dex, CpdA and ZK216348 on intestinal epithelial cell migration, a well-established *in vitro* wound healing model was performed, utilising the non-transformed rat small-intestinal epithelium cell line (IEC-6). After 24 h, the presence of Dex significantly impeded the migration of IEC-6 cells into the wounded area in a concentration-dependent manner, compared to control cells cultured in medium alone (Figure 4A). In contrast to Dex, CpdA and ZK216348 did not inhibit wound closure, (Figure 4B, 4C and 4E). Additional evidence that SEGRAs do not exert a negative influence on cell migration was gained using the skin derived keratinocyte cell line HaCaT was utilised in the same *in vitro* wound healing model. Results were similar to those attained using IEC-6 cells, indicating that, while Dex, concentration-dependently inhibits wound closure, this is not the case with CpdA or ZK216348 (Table 1).

**Table 1:** Effect of SEGRAs on HaCaT cell restitution.

Dex	wound closure	CpdA	wound closure	ZK216348	wound closure
[µM]	[%]	[µM]	[%]	[µM]	[%]
**-**	65.89±2.64	**-**	66.0±2.38	**-**	63.82±2.31
**0.01**	61.63±3.03	**0.1**	67.06±4.15	**0.1**	58. 93±4.00
**0.05**	52.25±2.74 *	**1**	64.41±2.17	**1**	59.93±3.16
**0.1**	46.26±5.08 **	**5**	65.13±2.97	**5**	60.92±4.58
**0.5**	38.10±2.79 ***	**10**	61.21±3.48	**10**	62.14±4.34
**1**	27.80±1.64 ***	**20**	64.94±2.36	**20**	57.36±5.07

HaCaT cells were wounded and cultured in the presence of Dex, CpdA or ZK216348 for 24 h. Cell migration was assessed using an *in vitro* migration assay. Data indicate mean values of remaining wounded area ± S.E.M., n = 3,

*P≤0.05,

**P≤0.01,

***P≤0.001 relative to control.

### Effects of CpdA and ZK216348 on intestinal epithelial cell proliferation

After it was shown that Dex modulates intestinal epithelial migration, whereas SEGRAs do not, the effects of the three substances on epithelial cell proliferation, the subsequent step to migration in wound healing, was studied. After 24 h, Dex and ZK216348 showed no significant effect on BrdU-incorporation into DNA of IEC-6 cells at concentrations between 0.1 µM and 20 µM. While this was also true of CpdA in concentrations between 0.1–10 µM (Figure 4D) a decrease in cell proliferation was observed above 10 µM, which is in accordance with its examined apoptotic and cytotoxic effects in concentrations higher than 15 µM (see Figure 3). Nevertheless, it can be ruled out that the beneficial effects of CpdA, at least below 15 µM, and of ZK216348, seen in the *in vitro* wound healing assay, are due to substance influences on cell proliferation. Hence, within these *in vitro* assays, the novel SEGRAs displayed no negative influence on intestinal epithelial cell restitution and proliferation, a fact which, in combination with their anti-inflammatory properties, might give them the advantage over common synthetic GCs, as, along with the attenuation of inflammation, mucosal healing is defined as an important goal of IBD management [32].

### Effects of CpdA and ZK216348 on TGF-β - mediated intestinal epithelial cell restitution

**Figure 5:**
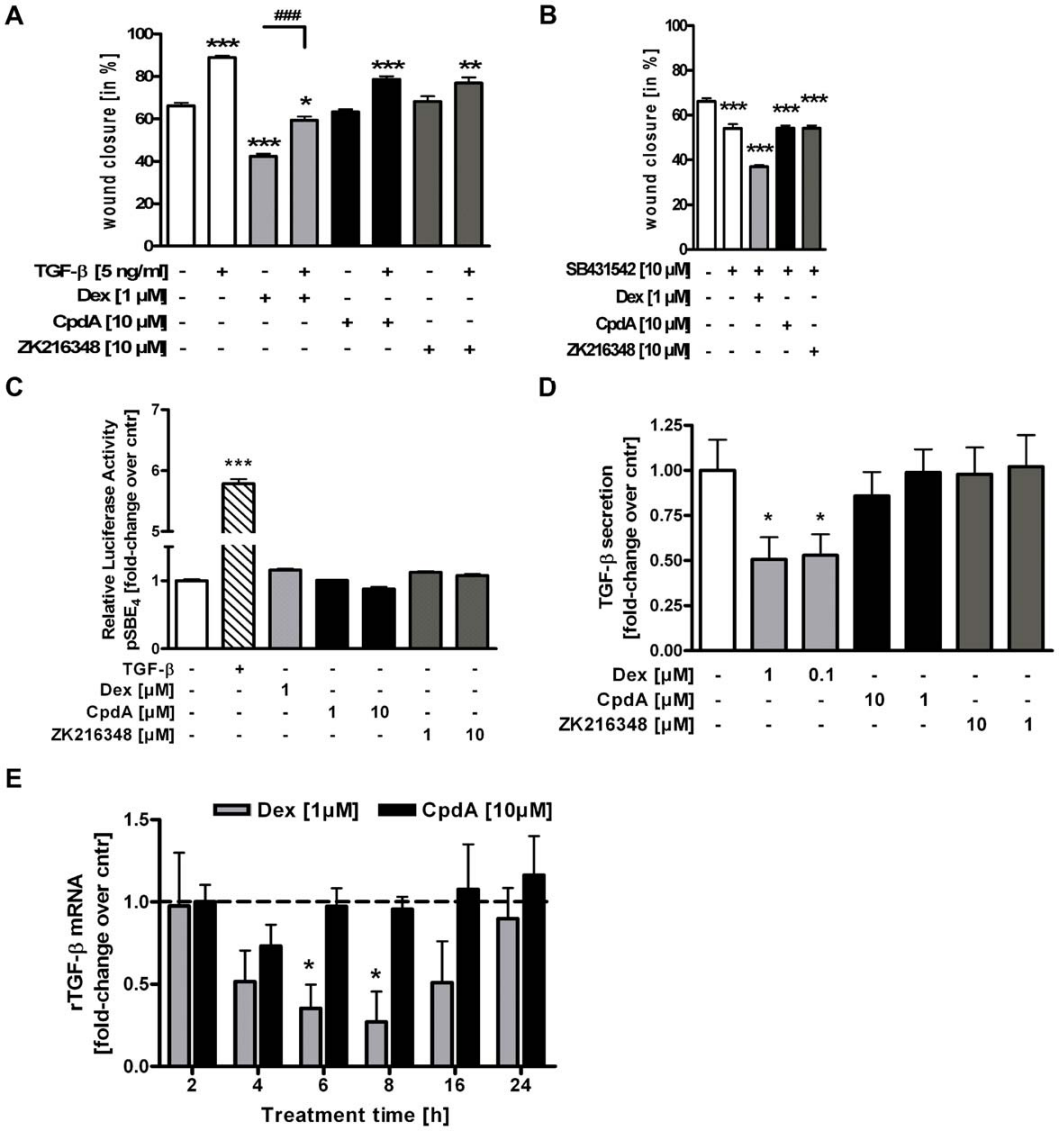
Effect of SEGRAs on TGF-β - mediated intestinal epithelial cell restitution. Wounded IEC-6 cells were cultured for 24 h in the presence of Dex, CpdA or ZK216348 with or without co-presence of (**A**) TGF-β or (**B**) selective inhibitor of activin receptor-like kinase (ALK) receptor SB431542. Cell migration was assessed using an *in vitro* migration assay. Bars indicate mean values of remaining wounded area ± S.E.M., n = 3, *P≤0.05, **P≤0.01, ***P≤0.001 relative to vehicle, ^###^ *P*≤0.001 relative to Dex (**C**) Relative luciferase activity of HEK293T cells transfected with the reporter gene construct pSBE4-luc construct after 24 h incubation with TGF-β (5 ng/ml), Dex or SEGRAs. IEC-6 cells were treated with Dex, CpdA or ZK216348 for the indicated time periods. (**D**) TGF-β peptide levels in cell culture supernatants were determined by ELISA. (**E**) TGF-β mRNA expression was monitored by qPCR and normalised against β-actin. Bars represent mean ± S.E.M., n = 3, *P≤0.05 relative to vehicle.

Several cytokines and chemokines expressed in the intestinal mucosa promote epithelial restitution after injury through increased production of bioactive TGF-β1 in epithelial cells. To check if Dex or SEGRAs modulate intestinal epithelial migration by TGF-β, or its pathway, the migration properties of IEC-6 cells in the presence or absence of exogenously-added TGF-β or SB431542, a selective inhibitor of activin receptor-like kinase (ALK) receptors, was investigated. As expected, both TGF-β alone and TGF-β in combination with CpdA or ZK216348 increased IEC-6 wound closure compared to vehicle treatment. Interestingly, TGF-β in the presence of Dex could only partially reverse the inhibitory effect of Dex on intestinal epithelial migration (Figure 5A). The blockade of TGF-β receptor brought about by the addition of SB431542 significantly decreased cell migration into the wounded area and further intensified the already inhibitory effect of Dex. In the co-presence of CpdA or ZK216348, however, no difference in epithelial cell migration was found in comparison to that seen in SB431543 treatment alone, suggesting a different modulation of the TGF-β-dependent promotion of cell migration (Figure 5B).

The binding of TGF-β to its receptor initiates the intracellular signalling machine via Smad proteins, and transcription is started by the binding of the smad3/4 complex to smad binding elements (SBE) in the promoter of TGF-β-sensitive genes. Indeed in HEK293T cells transiently transfected with a reporter gene construct carrying four SBEs (SBE4-Luc) the addition of TGF-β induced luciferase activity up to 5-fold, while neither the addition of Dex nor CpdA or ZK216348 showed any effect compared to vehicle control (Figure 5C). Although TGF-β peptide levels and mRNA expression (Figure 5D and E) were decreased after Dex-treatment, CpdA and ZK216348 revealed no modulatory effects, hinting toward a TGF-β-independent mechanism for unrestricted wound closure in the presence of SEGRAs.

### Effects of CpdA and ZK216348 on EGF/ERK1/2/MAPK pathway-mediated intestinal epithelial cell restitution

**Figure 6:**
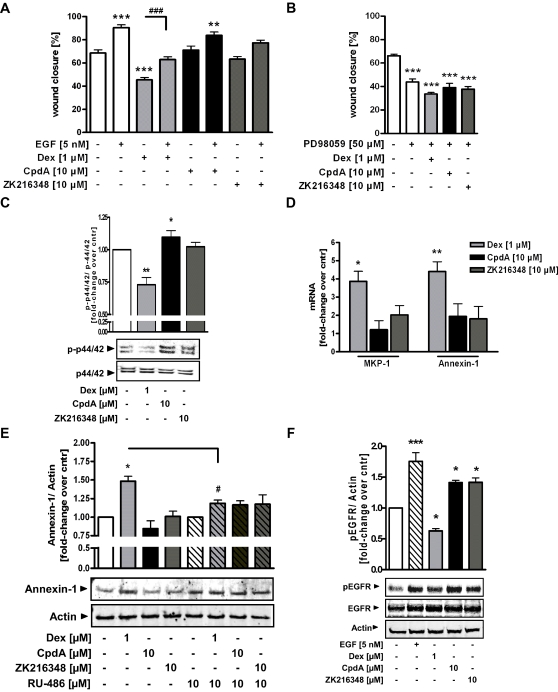
Effect of SEGRAs on EGF/ERK1/2/MAPK pathway - mediated intestinal epithelial restitution. Wounded IEC-6 cells were cultured for 24 h in the presence of Dex [1 µM], CpdA or ZK216348 [10 µM] with or without co-presence of (**A**) EGF (5 nM) or (**B**) specific inhibitor of MEK1/2 phosphorylation PD98059 [50 µM]. Cell migration was assessed using an *in vitro* migration assay. Bars indicate mean values of remaining wounded area ± S.E.M., n = 3, *P≤0.05, **P≤0.01, ***P≤0.001 relative to vehicle, ^###^ *P*≤0.001 relative to Dex. *(* ***C*** *)* Western blot analysis for pERK1/2 and ERK2 in Caco-2/GR cell lysates treated with Dex [1 µM], CpdA or ZK216348 [10 µM] for 1 h. One representative blot of three is shown and densitometric analysis of pERK1/2 is normalised to ERK2. (**D**) MKP-1 or Annexin-1 mRNA expression in Caco-2/GR cells after 6 h of treatment was monitored by qPCR and normalized against β-Actin. Western blot analysis for (**E**) Annexin-1 or (**F**) EGF-R protein expression in Caco-2/GR cells treated for 24 h or 1 h respectively with Dex or SEGRAs in the presence or absence of RU-486 [10 µM]. One representative blot of three is shown. Bars indicate mean ± S.E.M., n = 3, *P≤0.05, **P≤0.01, ***P≤0.001 relative to vehicle.

In addition to TGF-β, the extracellular stimulus EGF is also known as a strong enhancer of epithelial cell restitution [24], [33], [34] and triggers the ERK1/2/MAPK pathway, which is linked to cell migration and proliferation. Thus, the migration properties of IEC-6 cells in the presence or absence of exogenously added EGF and PD98059, a selective inhibitor of MEK1 phosphorylation upstream of ERK1/2, were examined, to see if Dex or the SEGRAs CpdA and ZK216348 modulate intestinal epithelial migration by this pathway. Indeed, EGF alone and in combination with CpdA and ZK216348, increased wound closure significantly. If cells were incubated with Dex in combination with EGF, EGF was able to compensate the inhibitory effect of Dex on intestinal epithelial migration that was observed with Dex alone (Figure 6A). Furthermore, the blockade of ERK1/2 phosphorylation by addition of PD98059 diminished wound closure, alone or in the co-presence of Dex, CpdA and ZK216348, underscoring the importance of this pathway in epithelial repair (Figure 6B).

GCs have been shown to interfere with the ERK1/2/MAPK pathway and amongst other a decreased ERK1/2 phosphorylation was observed. Western blot analysis of the ERK1/2 phosphorylation status of Caco-2/GR cells after treatment with Dex or SEGRA indeed revealed reduced ERK1/2 phosphorylation when cells were exposed to Dex, but not CpdA or ZK216348 (Figure 6C). It has been shown that this ERK1/2 phosphorylation is blocked by MKP-1, whose expression is typically initiated by the trans-activation mechanism of GCs [35], [36]. In comparison to vehicle-treated Caco-2/GR cells, Dex-treated cells revealed an up-regulation in MKP-1 mRNA levels, while CpdA and ZK216348, as SEGRAs, showed no significant induction of MKP-1 expression (Figure 6D). The same was true for expression of Annexin-1 mRNA and protein (Figure 6D and E), likewise a well-known trans-activated gene and competitor for the phosphorylation of EGF receptor (EGFR), which in turn was blocked in the presence of Dex but not SEGRAs (Figure 6F). Addition of the GR antagonist RU-486 reversed the Dex-mediated induction of Annexin-1, therefore indicating the GR-dependency of the increase of Annexin-1 expression (Figure 6E). These data suggest that the difference in the inhibitory effects of Dex and the SEGRAs CpdA and ZK216348 on wound closure might be a result of their different interference with the EGF/ERK1/2/MAPK pathway, again pointing towards a clear advantage for SEGRAs in IBD treatment.

## Discussion

Glucocorticoids are highly effective in combating inflammation, and represent a powerful tool in the management of inflammatory response in IBD patients [7], [37]. Unfortunately, their long-term use in particular causes a large number of debilitating side effects, thus restricting their application. Hence, in IBD therapy, there is a need for drugs, which are as effective as common GCs, but have a reduced side effect profile. This demand might be satisfied by the novel class of SEGRAs, following the promising concept of selective modulation of GR action. In the present *in vitro* study in intestinal epithelial cells, it has been demonstrated that the beneficial anti-inflammatory actions of the GR agonists CpdA and ZK216348 are comparable to those of the steroids' representative Dex. Strikingly, and in contrast to results observed with Dex, this was found in the absence of intestinal epithelial wound healing inhibition, a classical steroid-associated side effect, thus emphasizing SEGRAs' potential as possible future IBD therapeutics.

The efficacy of GCs in diminishing inflammation, results from the pleiotropic effects of their receptor (the activated GR) on multiple signalling pathways. Within this study, the SEGRAs CpdA and ZK216348 were confirmed as functional GR agonists in intestinal cells, as GR activation and transmigration to the cell nucleus occurred following application of CpdA and ZK216348. After translocation, several models for transcriptional modulation by the GR have been presented. It is widely accepted that the trans-repressive mechanism is responsible for the beneficial anti-inflammatory effect of GCs, while their side effects are thought to result from the transcriptional activation of genes. Indeed, within this study CpdA and ZK216348 have shown desirable trans-repressive, and therefore anti-inflammatory, action in intestinal epithelial cells by inhibiting the activity of NF-κB, a transcription factor associated with numerous pro-inflammatory genes in IBD [38]. Moreover, the expression of the NF-κB-driven cytokine IL-8 in TNF-α-/IL-1β-stimulated cells was significantly reduced and the anti-inflammatory action of the novel SEGRAs, CpdA and ZK216348 found to be comparable to that of Dex. Furthermore, both SEGRAs induced trans-activation of GRE-containing promoter-reporter constructs to a lesser extent than Dex, and expression of the typically GRE-driven genes, MKP-1 and Annexin-1, was induced neither by CpdA nor ZK216348, leading to the conclusion, that in intestinal epithelial cells also, CpdA and ZK216348 show dissociative properties attributed to the class of SEGRAs.

Previous studies applying CpdA and ZK216348 *in vitro* and *in vivo* focused on several side effects associated with GC-treatment such as GC resistance [39] or hyperglycaemia [13], [14], [15], [16], [40]. In addition to these side effects, GCs are known inhibitors of wound healing [20], [29], [41], which considerably limits their therapeutic application in various inflammatory conditions. The mechanism behind the inhibitory effect of GCs on tissue repair is mainly subscribed to the ability of GCs to regulate the expression of several key proteins at the wound site [29], but the modulation of a range of other physiological processes such as metabolism, migration, cell proliferation and differentiation also plays an important role [18], [19], [41].

In the context of IBD, where the continuity of the mucosal epithelial surface constitutes a key requisite for combating the inappropriate and ongoing activation of the immune system [20], [42], impaired tissue repair under GC therapy represents a very drastic side effect. In the present study, a dose-dependent inhibition of intestinal epithelial restitution by Dex was observed, consistent with results described for budesonide and prednisolone [18], [19]. Strikingly, neither CpdA nor ZK216348 showed negative effects on intestinal epithelial migration or proliferation. Thus, a beneficial effect for SEGRAs in wound healing can be stated. This is further supported by the absence of inhibitory effects on migratory capacity of the skin-derived keratinocyte cell line HaCaT. Moreover, the present results confirm the conclusion drawn by Grose et al., who studied the role of endogenous GCs in wound repair in mice harbouring the DNA-binding-defective mutant version of the GR (GR^dim^), and observed improved wound healing compared to wild type mice. While unfamiliar with the class of SEGRAs, the authors suggested the use of more specific GCs as advantageous therapeutic modalities for wound healing [43].

Further experiments were conducted to identify the differentially modulated mechanism of GCs and SEGRAs in intestinal wound healing. A central role has been highlighted for the wound healing promoting factor TGF-β, as many of the regulatory peptides have been demonstrated to enhance epithelial restitution through a TGF-β-dependent mechanism. Additionally, the improved wound healing seen in GR^dim^ mice, could be linked to enhanced fibroblast secretion of TGF-β [43]. It therefore seems conceivable that the unrestricted intestinal wound closure observed under treatment with the SEGRAs CpdA and ZK216348 might be due to a different modulation of TGF-β or its signalling pathway. However, in line with previous studies using the GCs prednisolone and budesonide, addition of TGF-β only partially reversed the Dex-mediated inhibition of cell restitution, and other than an inhibitory effect of Dex on TGF-β levels in IEC-6 cells, no significant changes in the TGF-β signalling pathway for CpdA or ZK216348 could be found.

This implicates the involvement of a non-Smad TGFβ-induced signalling mechanism in epithelial wound closure that complements the canonical Smad pathway and might be differently modulated by GCs and SEGRAs. With respect to mucosal healing, one of the regulatory peptides, EGF, likewise a strong enhancer of epithelial cell restitution [24], [33], [34], and its signalling via the EGF/ERK1/2/MAPK pathway, are of particular interest, as its downstream targets are involved in cellular events such as cell motility and proliferation [28], [44], and the influence of GCs upon the EGF/ERK1/2/MAPK pathway is also well documented [45], [46]. Indeed, addition of exogenous EGF in combination with Dex led to a complete restoration of the Dex-mediated decrease of restitution in the utilised *in vitro* wound healing model. Also, in correlation with the ERK1/2 and EGFR phosphorylation status expression of MKP-1 and Annexin-1 was significantly induced by Dex but not by CpdA or ZK216348. Taken together, these findings indicate the involvement of the EGF/ERK1/2/MAPK signalling cascade in the Dex-mediated inhibition of intestinal epithelial wound closure *in vitro*. More important, however, is the fact that neither CpdA nor ZK216348 induce the expression of MKP-1 and Annexin-1, and thus in their presence the signalling cascade is still active and wound healing promoted. These data again match the study in GR^dim^ mice, in which improved wound healing in comparison to wild type mice suggests that the inhibitory effect of exogenous GCs is at least to some extent mediated by the DNA binding activity of the GR (trans-activation mechanism) [43].

The ability of CpdA and ZK216348 to dissociate between trans-repression and trans-activation in various inflammatory models has been shown previously, but for the first time this is here demonstrated in the context of IBD, where it has successfully been shown that not only do these SEGRAs have anti-inflammatory properties, but in addition, they do not negatively interfere with the process of intestinal epithelial wound healing. However, for future therapeutic use, a close characterisation of SEGRAs pharmacology and mode of action is mandatory, e.g. with regard to substance safety, as the apoptotic and cytotoxic potential of CpdA in particular results in a narrow therapeutic window [31]. Nevertheless, CpdA and ZK216348 are suitable model substances and we suggest that the concept of SEGRAs offer a promising perspective in the treatment of IBD, where a strong preference is given to inflammatory gene repression, and where reduction of GC-linked side effects, e.g. impaired wound repair (mucosal healing) is of considerable importance.

## Materials and Methods

### Cell culture and Materials

IEC-6, HeLa, Caco-2, HEK293T and HaCaT cells were purchased from the Deutsche Sammlung für Mikroorganismen und Zellkulturen (DSMZ, Braunschweig, Germany) and maintained in Dulbecco's modified Eagle medium (DMEM, Invitrogen) with 10% fetal calf serum (FCS, PAA) 100 U/ml penicillin and 100 mg/ml streptomycin (PAA). For cultivation of Caco-2, this medium was supplemented with 1% sodium pyruvate (PAA). HaCaT cells were kept in DMEM high glucose (Invitrogen) with 10% FCS, 100 U/ml penicillin, 100 mg/ml streptomycin and 2 mM Glutamine (PAA). Stock solutions were prepared by dissolving Dex (SIGMA-Aldrich) in water, CpdA (Alexis Biochemicals) in PBS (pH 6.0), ZK216348 (kindly provided by Dr. Schäcke, Bayer Pharma AG, Berlin) in DMSO, all at a concentration of 10 mM. RU-486 (SIGMA-Aldrich), SB431542 and PD98095 (Calbiochem) were dissolved in DMSO at 10 mM. For experiments, the cells were seeded on plastic cell culture wells and allowed to attach for 24–48 h. Prior to Dex or SEGRA stimulation, cells were cultured in medium containing 1% FCS for 16 h. Dex, SEGRAs and inhibitors, whenever used, were given in pre-incubations 1 h before addition of other reagents. For subsequent stimulation, recombinant human TNF-α, IL-1β, EGF and TGF-β (all PreproTech) were dissolved in water/1% bovine serum albumin (BSA) and added to the medium to their final concentrations. Unless otherwise stated, all chemicals were obtained from Sigma-Aldrich.

### Cloning of pGR-FL & pGRE-Luc backbone

An expression plasmid for the human full-length glucocorticoid receptor (pGR-FL) was generated by cloning GR from HepG2 genomic DNA by PCR using the following primers: GR-FL-for: 5′-CGCGGATCCATGGACTCCAAAG-3′, GR-FL-rev: 5′-CGCTCTAGATCACTTTTGATGAAACAG-3′ (Eurofins). The resulting PCR fragment was inserted in the pCDNA3 vector (Invitrogen) after its digestion with BamHI and XbaI enzymes (Fermentas). The functionality of GR expression was monitored by Western blot. For the pGRE-Luc backbone construct, the GRE-enhancer was removed by digestion of pGRE-Luc (reporter gene construct for GRE-driven luciferase expression, kindly provided by Prof. Schulzke, Berlin) with NheI and BglII. A custom-made phosphorylated linker lacking GREs (Eurofins; Forward: 5′ – CTAGCATCGGATCA -3′, Reverse: 5′- GTAGCCTAGTCTAG -3′) was inserted before the P_TAL_-Promoter region.

### Transfection and Reporter Gene Assay

Caco-2 cells were transiently transfected with the GR expression plasmid pGR-FL alone (200 ng, referred to as Caco-2/GR) or like HEK293T cells, together with the indicated amount of reporter gene constructs using Lipofectamine 2000 reagent (Invitrogen) according to manufacturer's instructions. Briefly, for the reporter gene assay, cells were seeded in 24-well plates in cell line specific medium without phenol red. The following plasmid concentrations were used for transfection: 100 ng pGR-FL, 700 ng pGRE-Luc and 50 ng pSV-40 Renilla as internal standard for Caco-2, 200 ng of the reporter gene construct pSBE_4_-Luc containing 4×SBE (Smad binding elements, generously provided by B. Vogelstein, Baltimore, USA) and 50 ng pSV-40Renilla for HEK293T. The medium was changed after 5 h of transfection and cells were incubated for another 16 h before being treated as indicated. Reporter gene activity was assayed with the Dual-Luciferase Reporter Assay System (Promega) and a TECAN infinite® M200 Luminometer. Data are shown as relative light units (RLU) as a percentage of control, normalised to transfection efficiency (co-transfection of pSV-40-renilla) and normalised to effects of the respective empty vectors (pCDNA3, pGRE-Luc backbone construct, pGL3basic or pCGN).

### Immunofluorescence

Caco-2 cells plated on sterile chamberslides were treated for 3 h with Dex, SEGRAs or vehicle. The cells were fixed for 20 min with 40% aceton/60% methanol at −20°C and kept in blocking buffer (3% BSA/PBS/0.1% Tween-20) for 30 min at room temperature. Subsequent incubation with anti-GR (SantaCruz Biotechnology) diluted in blocking buffer for 30 min at 37°C, and three washing steps with 0.1% Tween-20/PBS/0.1% BSA, secondary antibody Cy™3-conjugated goat-anti-rabbit IgG (Zymed) diluted in blocking buffer was applied for 30 min at room temperature. The slides were counterstained with 4′,6-Diamidino-2-phenylindole (DAPI, Vector Laboratories) mounting medium after another washing and analysed using an Olympus IX71 microscope at 100×magnification. To quantify translocation, 100 randomly-chosen cells per treatment were analysed and processed using GSA Image Analyzer. The percentage of GR translocation was calculated by dividing the area of pink (merge of Cy™3 and DAPI) by blue fluorescence (DAPI) per field.

### Electrophoretic mobility shift assay (EMSA)

Consensus oligonucleotide for NF-κB (sc-2505) was obtained from SantaCruz Biotechnology and endlabeled by polynucleotide kinase (PNK) using a-ATP (3000 Ci/mM). Binding reactions were performed for 30 min on ice with 5 µg of IEC-6 nuclear extract protein as described previously [47]. Polyclonal antibodies used for supershift experiments were purchased from SantaCruz Biotechnology and were added 15 min after addition of the labelled probe and co-incubated on ice for a further 30 min.

### Cytoplasmic/nuclear extract preparation and NF-κB activation assay

Cytoplasmic and nuclear extracts from IEC-6, Caco-2 or HeLa cells were prepared using the nuclear extraction kit from Active motif (Active motif) following the manufacturer's instructions. Nuclear NF-κB/p65 activity was measured with TransAM® NF-κB p65 kit (# 40096, Active motif).

### Protein extract preparation and Western blot analysis

For the isolation of whole cell extracts, cells were harvested at 4°C in Cell lysis buffer (Cell Signaling) containing multiple protease inhibitors (Complete Mini®, Roche). Soluble protein extracts were obtained after sonication of crude lysates and centrifugation at 10 000 rpm for 10 min at 4°C. Samples were separated by sodium dodecylsulfate – polyacrylamid gelelectrophoresis (SDS-PAGE) and transferred onto nitrocellulose membranes (Hybond C, Amersham) following standard protocols. After blocking, membranes were incubated overnight at 4°C with the indicated primary antibodies: anti-GR (sc-8992), anti-p65 (sc-372), anti-IκB-α (sc-371), anti-EGFR (sc-03) and anti-pEGFR (sc-12351) (all from SantaCruz Biotechnology) and anti-p42/44 (#4695), anti-p-p42/44 (#4377S) and anti-Annexin-1 (#3299S) (all from Cell Signalling) followed by infrared dye-conjugated secondary antibodies (LI-COR® Biosciences). Bound complexes were detected with the Odyseey® infrared imaging system (*LI-COR*®). For quantification, the intensities of the total protein were normalised to β-actin (#A2228, SIGMA-Aldrich) or lamin (#2032, Cell Signaling) signal.

### Apoptosis Assay

IEC-6 cells were incubated for 24 h in the presence or absence of Dex, CpdA or ZK216348 and harvested with 1%-Trypsin-EDTA (PAA), washed twice with ice-cold PBS, and suspended in binding buffer as instructed by the manufacturer (Apoptosis detection kit #559763, BD Biosciences). Aliquots of cells (100 µl) were incubated with Annexin V/7-AAD and 100 µl counting beads (CALTAG™ Counting beads; Invitrogen). Data acquisition (10 000 beads) and analysis were performed in a FACS analyser (FACSCanto, BD Biosciences).

### Caspase-3 activity assay

Caco-2 cells were seeded in 6-well dishes and allowed to reach confluency. Caspase-3 activity was analysed after 24 h of incubation with Dex or SEGRAs using a fluorometric immunosorbent enzyme assay (#12 012 952 001, Roche) according to the manufacturer's instructions with the following modification: All cells per treatment were harvested and cell lysate used for Caspase-3 activity assay. After fluorometrical determination of free fluorescent AFC, total protein concentration of the samples was measured and adapted to the activity to correct for variation in protein concentration.

### Cytotoxicity and Cell proliferation assay

Cells were cultured at a density of 0.01×10^6^ cells in 96-well dishes, allowed to attach over night and treated for 24 h with different concentrations of Dex or SEGRAs as indicated. Cytotoxicity was determined by measuring lactate dehydrogenase (LDH) release using a commercially available kit (Cytotoxicity detection kit (LDH), Roche) following the manufacturers instructions. Proliferation of IEC-6 cells was determined by analysing 5′-Bromo-2′-Deoxy-uridine (BrdU) incorporation into newly synthesised DNA using a cell proliferation enzyme linked immunosorbent assay (ELISA) (Cell proliferation ELISA (BrdU), Roche).

### Migration (Restitution) Assay

An epithelial cell wound healing model was performed using a modified version of the previously described techniques [18], [48]. Confluent monolayers of IEC-6 or HaCaT cells were wounded in a standardised procedure. Three independent wounds (∼20–25 mm, horizontal) per dish were established with a sterile pipette tip and areas marked with a sterile razor blade (vertical). After scraping, cells were washed with fresh medium and wounds photographed (Sony, DSC-S75) at 100-fold magnification (Axiovert 135, Carl Zeiss) using an ocular reticle, which allowed clear recovery of the photographed area. Cells were cultured for a further 24 h in fresh, serum-deprived medium in the presence or absence of Dex or SEGRAs, individually or in combination with EGF, TGF-β, SB431542 or PD98059. Hereafter, wound areas were photographed again and migration of cells was assessed by comparing the calculated (WEGA-Image Viewer, M.O.S.S.) wound area before and after 24 h. At least 8 wounded areas per dish were analysed and three independent experiments performed.

### IL-8 and TGF-β ELISA

Cells were seeded in 6-well dishes and treated as described. The concentration of Interleukin 8 (IL-8) in the supernatants was measured by commercially available Quantikine® ELISA kit (R&D Systems). In IEC-6 supernatants for the determination of total (latent plus bioactive) TGF-β, cell culture supernatants were first activated by acidification with 1 N HCl for 10 min, followed by neutralisation with 1.2 N NaOH/0.5 M HEPES at room temperature. TGF-β concentration was determined using the rat TGF-β Quantikine® ELISA (R&D Systems). The ELISAs were performed according to the manufacturer's instructions.

### Isolation of Total RNA and qPCR

For isolation of total RNA, cells were purified with TRIzol® (Invitrogen) according to the manufacturer's instructions. 2 µg of total RNA underwent DNAse I digestion (Fermentas) and subsequently were subsequently reverse transcribed using the iScript cDNA Synthesis kit (Bio-Rad). Real-time PCR for gene expression of MKP-1, Annexin-1 or rTGF-β was performed in cDNA (diluted 1 ∶ 5) using the Power SYBR Green PCR master mix with the AB StepOnePlus (Applied Biosystems) Sequence Detector system under the standard protocol. Human or rat β-Actin was used to normalise the results. The relative mRNA expression of each studied gene was calculated with the comparative ΔCt method using the formula 2^−ΔΔCt^.

### Statistics

The data are expressed as means ± S.E.M. Analysis of variance (ANOVA) was performed when more than two groups were compared, and when significant (*P*<0.05), multiple comparisons were performed with the Tukey test. A *P* value≤0.05 was considered to be significant.

## Supporting Information

Figure S1 **Comparison of trans-activation and trans-repression effects of SEGRAs in different concentrations.** (**A**) Relative Luciferase Activity of Caco-2/GR cells transfected with the glucocorticoid response element (GRE)-driven luciferase construct (pGRE-luc) and pSV-40 Renilla after 24 h of treatment with or without Dex [1–50 µM], CpdA or ZK216348 [1–50 µM]. (**B**) Caco-2 cells were pre-treated with Dex [0.1–10 µM], CpdA or ZK216348 [1–50 µM] for 1 h before 15 min cultivation in co-presence or absence of TNF-α [0.5 nM] and harvesting for nuclear protein extract preparations. NF-κB activity was measured by transcription factor assay for p65. Bars indicate mean ± S.E.M., n = 3, *P≤0.05, **P≤0.01, ***P≤0.001 relative to vehicle or TNF-α, respectively. **Concentration-dependent trans-repression and –activation effects of CpdA and ZK216348:** Clearly, each of the three GR-agonists has a different potency and therefore the pGRE-reporter gene assay was used to test their trans-activation activity over a wider range of concentrations. Cell treatment with higher concentrations of Dex resulted in dose-dependent acceleration of relative luciferase activity (Figure S1A). This was also observed for ZK216348 treatment (Figure S1A), which one could already suspect from the data obtained and pictured in Figure 2 E. No induction of luciferase activity was observed at higher CpdA concentrations (Figure S1A), a result most likely attributable to CpdA's cytotoxicity and apoptosis inducing properties above 20 µM (Figure 3). Both firefly and Renilla values were much lower in concentrations >20 µM, so that after normalization, CpdA would appear to have less trans-activation potential than Dex or ZK216348 (Figure S1A). Similarly, employing the TransAM® p65 Kit for NF-κB activation after cytokine stimulation in Dex- or SEGRA-treated Caco-2 cells, no potentiating effect of CpdA could be shown for evidently cytotoxic or apoptotic concentrations. However, the treatments of cells with increasing concentrations of ZK216348 lead to an increased inhibition of NF-κB activity (Figure S1B). In summary, ZK216348 and especially CpdA, with its additional apoptotic potential, possess a narrow therapeutic window in which they act as “dissociating” GR ligands, i.e. suppressing inflammatory effects without displaying GR-mediated trans-activation.
(TIF)
