# Supplementary figures and images for: Selective Non-Steroidal Glucocorticoid Receptor Agonists Attenuate Inflammation but Do Not Impair Intestinal Epithelial Cell Restitution In Vitro
Source: PLoS One. 2012 Jan 25;7(1):e29756. doi: 10.1371/journal.pone.0029756 (PMC3266253; doi:10.1371/journal.pone.0029756)

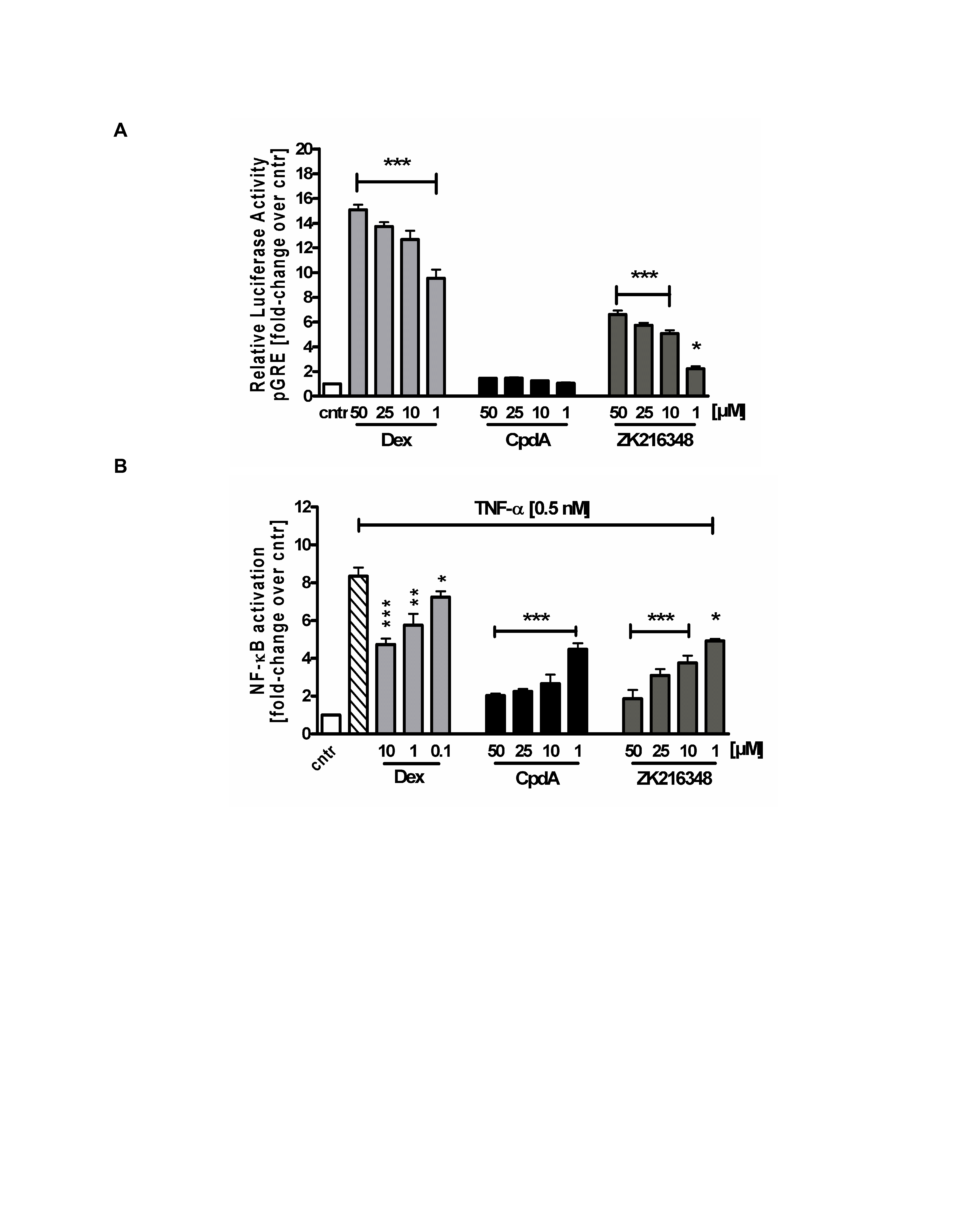

Supplement: Figure S1 — Comparison of trans-activation and trans-repression effects of SEGRAs in different concentrations. (A) Relative Luciferase Activity of Caco-2/GR cells transfected with the glucocorticoid response element (GRE)-driven luciferase construct (pGRE-luc) and pSV-40 Renilla after 24 h of treatment with or without Dex [1–50 µM], CpdA or ZK216348 [1–50 µM]. (B) Caco-2 cells were pre-treated with Dex [0.1–10 µM], CpdA or ZK216348 [1–50 µM] for 1 h before 15 min cultivation in co-presence or absence of TNF-α [0.5 nM] and harvesting for nuclear protein extract preparations. NF-κB activity was measured by transcription factor assay for p65. Bars indicate mean ± S.E.M., n = 3, *P≤0.05, **P≤0.01, ***P≤0.001 relative to vehicle or TNF-α, respectively. Concentration-dependent trans-repression and –activation effects of CpdA and ZK216348: Clearly, each of the three GR-agonists has a different potency and therefore the pGRE-reporter gene assay was used to test their trans-activation activity over a wider range of concentrations. Cell treatment with higher concentrations of Dex resulted in dose-dependent acceleration of relative luciferase activity (Figure S1A). This was also observed for ZK216348 treatment (Figure S1A), which one could already suspect from the data obtained and pictured in Figure 2 E. No induction of luciferase activity was observed at higher CpdA concentrations (Figure S1A), a result most likely attributable to CpdA's cytotoxicity and apoptosis inducing properties above 20 µM (Figure 3). Both firefly and Renilla values were much lower in concentrations >20 µM, so that after normalization, CpdA would appear to have less trans-activation potential than Dex or ZK216348 (Figure S1A). Similarly, employing the TransAM® p65 Kit for NF-κB activation after cytokine stimulation in Dex- or SEGRA-treated Caco-2 cells, no potentiating effect of CpdA could be shown for evidently cytotoxic or apoptotic concentrations. However, the treatments of cells with increasing concentrations [file pone.0029756.s001.tif]
